# How does digital infrastructure affect residents' healthcare expenditures? Evidence from Chinese microdata

**DOI:** 10.3389/fpubh.2023.1122718

**Published:** 2023-05-04

**Authors:** Huichao Han, Chenxi Hai, Tianqi Wu, Nianchi Zhou

**Affiliations:** School of Business, Nanjing University of Information Science & Technology, Nanjing, China

**Keywords:** healthcare expenditure, digital infrastructure, Broadband China, commercial insurance availability, residents' self-rated health

## Abstract

Healthcare expenditure is only one of the heavy burdens that families face in developing countries. Current research mainly focuses on analyzing the effects of financial policy. There is a lack of studies that examine the understanding and assessment of the impact of digital infrastructure on this issue. In this study, we used the Broadband China policy as a quasi-natural experiment to explore the impact of digital infrastructure on residents' healthcare expenditures in China. Using the differences-in-differences (DID) model and micro-survey data, we found that digital infrastructure has a positive impact on reducing the burden of healthcare expenditure in China. Our findings indicate that residents in cities can save up to 18.8% on healthcare expenses following large-scale digital infrastructure construction. Through mechanism analysis, we found that digital infrastructure reduces residents' healthcare expenditures by improving both commercial insurance availability and the healthcare efficiency of residents. In addition, the effects of digital infrastructure on reducing healthcare expenditure are more pronounced among middle-aged individuals, those with low levels of education, and those with low incomes, which indicates this digital construction wave helps bridge the social gap between the poor and the rich. This study provides compelling evidence of the positive impact of digital society construction on social health and wellbeing.

## 1. Introduction

Due to environmental pollution, an aging society, and the COVID-19 pandemic, healthcare expenditure is increasing in China and globally. According to World Health Organization (WHO) statistics, more than 500 million people have been pushed into extreme poverty due to the heavy burden of healthcare costs. This situation often leads to a decline in health status and decreased productivity among poorer groups (1, 2). The excessive burden of healthcare expenditures has a negative impact on human wellbeing, especially in developing countries. As the world's largest developing country, China faces various challenges, such as insufficient healthcare resources, uneven distribution of healthcare resources, severe air pollution, and an increasingly aging society, all of which contribute to the escalation of healthcare expenditure (3). In 2021, the average healthcare expenditure per capita was 5,348 RMB, which accounted for 8.8% of total personal expenditures. Although government healthcare spending continues to increase, it has not effectively reduced the individual healthcare burden, especially for rural residents and low-income groups (4). In 2018, the average individual healthcare share of expenditure in China was 35.8%, which is significantly higher than the levels in the US (10.8%) and Japan (12.8%). Hence, reducing healthcare expenditures is crucial for promoting universal health coverage and achieving sustainable development goals (SDGs) in China.

Digital technology is widely considered a potential solution to alleviate the pressure on healthcare systems (5–7). In recent years, significant improvements in digital infrastructure have driven a deeper integration of digital networks within the realm of healthcare, thereby facilitating access to health and medical care information (8). Current studies have found that digital technology not only helps patients recover but also effectively mitigates the rise of chronic diseases (9). In addition, the widespread use of digital technology plays an important role in improving air quality (10, 11), which helps reduce medical and defensive expenditures for air pollution-induced diseases (12–15). Therefore, digital technology has the potential to alleviate the burden on healthcare expenditures, even though there is no direct causal relationship between the construction of digital infrastructure and healthcare expenditures. Therefore, assessing the social welfare effect of digital infrastructure is crucial.

Previous studies have researched the relevant factors of healthcare expenditure from different perspectives. The first type of study examines the effects of financial policies, such as medical insurance, medical assistance, and pensions, on reducing residents' healthcare expenditures (16, 17). Ma et al. found that access to a new rural social pension significantly reduced the proportion of individual medical expenses (18). The second category of study extensively discusses the impact of environmental pollution on healthcare expenditures. A number of studies have confirmed that environmental pollution increases healthcare expenditures (12, 19–21). Xia et al. (22) found that both higher air pollution levels and longer-duration pollution events significantly increased healthcare expenditure. Liao et al. (23) used microdata to quantify the effect of air pollution on healthcare expenditures. Third, socioeconomic factors such as industrial agglomeration and education level may also influence the healthcare expenditure of residents (24–26). Li et al. (27) found that a higher level of education significantly reduces the occurrence of catastrophic medical expenses.

In summary, the existing literature rarely explores healthcare expenditure from the perspective of technological change. Only a few studies have examined the impact of Internet applications on residents' healthcare expenditures (28). Moreover, most studies in the literature have failed to address endogeneity issues, such as omitted variables, which can create causal problems between residents' healthcare expenditure and the factors that influence it in current research.

This study attempted to bridge this gap by taking the Broadband China Pilot Policy as a quasi-natural experiment to assess the impact of digital infrastructure construction on residents' healthcare expenditures. The method has been proven to be an effective way of addressing endogeneity problems. We utilized unbalanced panel data by matching the 2010–2018 microdata of the China Family Panel Studies (CFPS) with Broadband China at the city level. Furthermore, we incorporated individual, household, and city-level characteristics associated with digital infrastructure to account for the effects of underlying factors on healthcare expenditure. This study provided two mechanisms: digital infrastructure reduces residents' healthcare expenditures by improving commercial insurance availability, and it also improves healthcare efficiency for residents, which, in turn, reduces the healthcare burden.

This study contributed to the current literature as follows. First, based on both individual-level microdata and city-level data, we examined the impact of digital infrastructure construction on healthcare expenditure from the perspective of technological change. Few studies have examined the direct healthcare effects of digital infrastructure construction, especially in the Chinese context. Second, to address the potential endogenous problem in the empirical study, we used the differences-in-differences (DID) method and employed multi-year unbalanced panel data with time and province fixed effects, controlling for individual, household, and city characteristics to reduce the bias of the estimates. Finally, as studies exploring the potential mechanisms of the impact of digital technology on healthcare expenditure are relatively limited, we identified two underlying mechanisms that explain the impact of digital infrastructure on healthcare expenditure.

The rest of the article is organized as follows. Section 2 provides a policy background. Section 3 introduces empirical model variables and data specifications. Section 4 presents the estimation results for the digital infrastructure on healthcare expenditure and a series of robustness tests, mechanism analyses, and heterogeneous effect studies among different demographic groups. Section 5 concludes the article.

## 2. Policy background

Since the 1990s, China has been promoting its broadband network coverage and enhancing information transmission speeds. However, despite significant progress, China still lags behind Western countries in terms of digital infrastructure. To accelerate China's digital construction, in August 2013, China launched the *Broadband China Strategy and Implementation Plan* (hereinafter referred to as “Broadband China” for abbreviation). The purpose of Broadband China was to select the pilot cities that would receive significant investments in digital infrastructure from the central and regional governments. The first batch of 39 pilot cities was named in 2014, with the second and third batches of cities later selected in 2015 and 2016, respectively. Since the implementation of the Broadband China strategy, China has made significant progress with respect to digital infrastructure. In 2020, fixed broadband access capacity in Chinese cities had generally exceeded 100 Mbps, and fiber-optic broadband had been made available to over 98% of villages. The proportion of fiber-access users in China had reached 93.2%, which is significantly higher than the OECD average level of 26.8%. Hence, it is fair to conclude that China is leading the world in digital infrastructure construction. Not only has China constructed the biggest 4G networks, but it is also expanding 5G networks. The implementation of the Broadband China strategy has significantly improved the level of China's digital infrastructure. Consequently, the Broadband China strategy provides a rare opportunity for quasi-natural experimentation to assess the profound socioeconomic impact of digital infrastructure construction.

## 3. Methods

### 3.1. Methodology

In this study, we used the launch of the Broadband China strategy as a quasi-natural experiment to examine the impact of digital infrastructure on residents' healthcare expenditures. Considering that the policy of Broadband China was implemented in different years, we referred to Beck et al. (29) and constructed a time-varying DID model.


(1) $$\begin{array}{l} lnHE_{i,j,t}=\alpha_{0}+\alpha_{1}Policy_{i,j,t}+\sum \gamma_{j}X_{i,j,t}+\mu_{i}+\eta_{j}+\upsilon_{t}+\varepsilon_{i,j,t} \end{array}$$

where ln *HE*_*i, j, t*_ represents the healthcare expenditure of individual *i* in city *j* in year *t*; *Policy*_*i, j, t*_ represents the Broadband China Pilot Policy; *X*_*i, j, t*_ represents a set of control variables, μ_*j*_ represents city fixed effects, ε_*i, j, t*_ represents the residual term, υ_*t*_ represents the fixed effect of the year, α_0_ is the constant term, and α_1_ and y*j* are the variable coefficients. This study evaluated the effect of the Broadband China Pilot Policy on healthcare expenditure by observing the significance and magnitude of the variable coefficient α_1_.

### 3.2. Measure and description of variables

We mainly used two kinds of data. The key microdata on residents' healthcare expenditures in this study was collected from the China Family Panel Studies (CFPS), a nationally representative longitudinal survey of communities, households, and individuals launched in 2010 by the Institute of Social Science Survey (ISSS) at Peking University in China. CFPS is committed to providing the academic community with the most comprehensive and highest-quality survey data on contemporary China. The data used in this study were from CFPS 2010–2018. To obtain accurate estimation results, we collected two types of data from CFPS, including respondents' individual characteristics and household characteristics. This study considered variables that may be relevant to residents' healthcare expenditures, including information on respondents and households. The CFPS consists of three main components: an adult database, a household database, and a community database. However, they are separate from each other. If we want to control for both individual and household-level variables, we need to merge them through unique household codes. In addition, the policy shocks we used are at the prefecture and city levels; thus, we also needed to merge the already-merged CFPS data with the city-level data through unique city codes.

We also used macrodata, which mainly included GDP per capita, expenditure on science and education, population density, urban green coverage, and the value-added of secondary industries for each prefecture-level city in China from 2010 to 2018. All macrodata are taken from the China Urban Statistical Yearbook.

The sample selection process for this study was as follows: First, we matched individual and household data in the CFPS by unique household codes to obtain the CFPS dataset. Then, we matched the CFPS dataset with the municipality data using the unique municipality code. With the previous processing, we obtained a dataset covering individual, household, and prefecture-level characteristics.

#### 3.2.1. Dependent variables

This study considered the Broadband China Pilot Policy to be a quasi-natural experiment and used it to measure digital infrastructure construction. The Ministry of Industry and Information Technology and the National Development and Reform Commission of China designated 119 Broadband China demonstration cities in 2014, 2015, and 2016. We adopted the form of policy using a dummy variable; the variable *Policy* equals 1 if the city *j* was selected as the pilot city from the year *t*. Otherwise, it equaled 0.

#### 3.2.2. Independent variables

We used CFPS survey data to determine the residents' healthcare expenditures. In the CFPS questionnaires, a special question was asked: “How much has your household spent on healthcare in the past year?” The expenditure was measured by the constant price. We considered the natural logarithm of the variable's value.

#### 3.2.3. Mechanism variables

We examined two mechanism variables. The first was the ease of purchasing commercial insurance. It required interviewees to answer, “How much does your family spend on commercial insurance?” The mechanism variable was coded as 1 if the interviewer had bought commercial insurance and 0 otherwise. The second mechanism variable was residents' health status, which was obtained from the CFPS questionnaire item: “Do you consider yourself to be in good health?” The answers ranged from 1 (worst health) to 5 (best health). To facilitate analysis, we recorded the response options from 1 to 3, with 1 indicating poor health, 3 indicating very good health, and 2 indicating average health.

#### 3.2.4. Controlling variables

Two types of variables were controlled for in the analysis. The first type comprised individual demographic characteristics, which mainly include age, Hukou (household registration), gender, years of education, marital status, smoking and drinking status, household income per capita, household water source, and family size. The second comprised prefecture-level characteristics., which include population density (PD), science and education expenses (RD), greenery rate (Green), industrial structure (Second), and GDP per capita (GDPP). We applied the natural logarithm of the variables of household income per capita, PD, RD, Green, Second, and GDPP.

### 3.3. Data sources and descriptive statistics

Broadband information about China's pilot cities is issued by the Ministry of Industry and Information Technology and the National Development and Reform Commission. Healthcare expenditure mechanism Variables and individual-level control data come from the China Family Panel Studies (CFPS). CFPS is a national survey program initiated in 2010 that collects data from 25 provinces in China, covering 95% of the Chinese population. The sampling method for CFPS is based on a multi-stage approach. The CFPS program collects data every 2 years and aims to investigate family and individual information on a range of topics, including economic status, educational background, work status, and physical and mental health. The remaining controls in prefecture-level cities are from the China City Statistical Yearbook.

We matched healthcare expenditure and individual demographic variables with Broadband China and prefecture-level variables for each year to obtain a valid unbalanced panel data sample from 2010 to 2018. Table 1 shows the descriptions of the data. It includes observations, the mean, the standard deviation, and the maximum and minimum values of the main variables.

**Table 1:** Descriptive statistics.

**Variables**	**Observations**	**Mean**	**S.D**.	**Min**	**Max**
HE	173,119	6.863	2.625	0	14.00
Policy	118,776	0.191	0.393	0	1
Age	173,893	45.88	17.27	16	110
Hukou	164,895	0.272	0.445	0	1
Gender	172,268	0.495	0.500	0	1
Education	168,057	7.277	4.826	0	23
Marriage	163,973	0.726	0.446	0	1
Smoke	161,652	0.284	0.451	0	1
Drink	135,298	0.144	0.351	0	1
Household water	176,539	0.660	0.474	0	1
Preincome	165,889	9.188	1.380	0.182	15.22
Family size	174,833	4.332	1.968	1	26
Status	158,392	2.916	1.018	1	5
PD	118,776	6.378	1.243	1.630	11.06
RD	118,776	10.35	1.390	7.116	15.53
Green	118,776	3.655	0.405	0.47	6.121
GDPP	118,776	10.56	0.571	8.773	12.16
Second	118,776	3.835	0.223	2.757	4.41
Insurance	176,539	0.405	0.491	0	1
Healthy	176,068	2.552	0.746	1	3

## 4. Results

### 4.1. Baseline regression results

The baseline results of the impact of digital infrastructure on healthcare expenditures are reported in Table 2. Two-way fixed effects were controlled for in the main analysis. Column 1 reports the estimations of the impact of digital infrastructure on healthcare expenditures without controls, and Columns 2–3 display the regression results of models with individual characteristic controls and characteristic city controls introduced step by step. The results show that the coefficients of digital infrastructure are negative and significant at the 1% level. In other words, the digital infrastructure significantly reduces healthcare expenditure, whether controlled variables are added or not. The healthcare expenditure of treatment groups is, on average, reduced by 18.8% more than that of control groups. Therefore, digital infrastructure has a significant impact on reducing residents' healthcare expenditures.

**Table 2:** Estimation results of the benchmark model.

**Variables**	**HE**		
	**(1)**	**(2)**	**(3)**
Policy	−0.121^***^ (0.030)	−0.147^***^ (0.037)	−0.188^***^ (0.039)
Age		0.003 (0.030)	0.002 (0.029)
Hukou		0.110 (0.077)	0.108 (0.077)
Gender		−0.456 (0.382)	−0.450 (0.381)
Education		−0.019 (0.012)	−0.019 (0.012)
Marriage		−0.045 (0.063)	−0.043 (0.063)
Smoke		−0.197^***^ (0.052)	−0.200^***^ (0.052)
Drink		−0.096^**^ (0.043)	−0.096^**^ (0.043)
Lifewater		−0.131^***^ (0.034)	−0.139^***^ (0.034)
Preincome		0.089^***^ (0.012)	0.087^***^ (0.012)
Familysize		0.179^***^ (0.011)	0.180^***^ (0.011)
Status		−0.009 (0.013)	−0.009 (0.013)
PD			0.002 (0.022)
RD			0.047^**^ (0.023)
Green			0.140^***^ (0.030)
GDPP			0.385^***^ (0.116)
Second			−0.026 (0.176)
Constant	5.878^***^ (0.436)	4.101^**^ (1.891)	−1.091 (2.113)
Individual FEs	Yes	Yes	Yes
Province FEs	Yes	Yes	Yes
Year FEs	Yes	Yes	Yes
Observations	116,364	78,601	78,601
R-squared	0.012	0.022	0.023

In parentheses are standard errors adjusted for heteroskedasticity and clustering by both individual and year. ^*^, ^**^, and ^***^ are significant at the levels of 10, 5, 1%, respectively.

### 4.2. Robustness tests

#### 4.2.1. Parallel trend tests

Adopting the difference-in-differences model hinges on passing a parallel trend test; that is, the trend in healthcare spending by residents in pilot and non-pilot cities of Broadband China is the same as before the policy was taken. Following the method of Lyu et al. (30), we used an event-study approach to estimate the dynamic treatment effects in Broadband China. The empirical model is as follows:


(2) $$\begin{array}{l} lnHE_{i,j,t}=\theta_{0}+\sum_{\tau =-1}^{\tau =-6}\theta_{\tau }pre_{i,j,t}+\theta_{1}dummy_{i,j,t}\\ \;+\sum_{\sigma =1}^{\sigma =5}\theta_{\sigma }post_{i,j,t}+\theta_{2}control_{i,j,t}+\mu_{i}+\eta_{j}+\gamma_{t}+\varepsilon_{i,j,t} \end{array}$$

where *lnME*_*i, j, t*_ represents residents' healthcare expenditure, and *pre* is a set of counterfactual dummy variables. If it is assumed that the pilot policy of Broadband China has changed from τ implemented in (τ = 2,012, 2,010), then *pre* = 1, for the other years *pre* = 0. Assuming that the pilot policy of Broadband China was implemented since the σ in the year of implementation, *post* = 1, in other years *post* = 0, *dummy* = 1 in the year of implementation of the Broadband China policy, otherwise *dummy* = 0 in other years.

**Figure 1:**
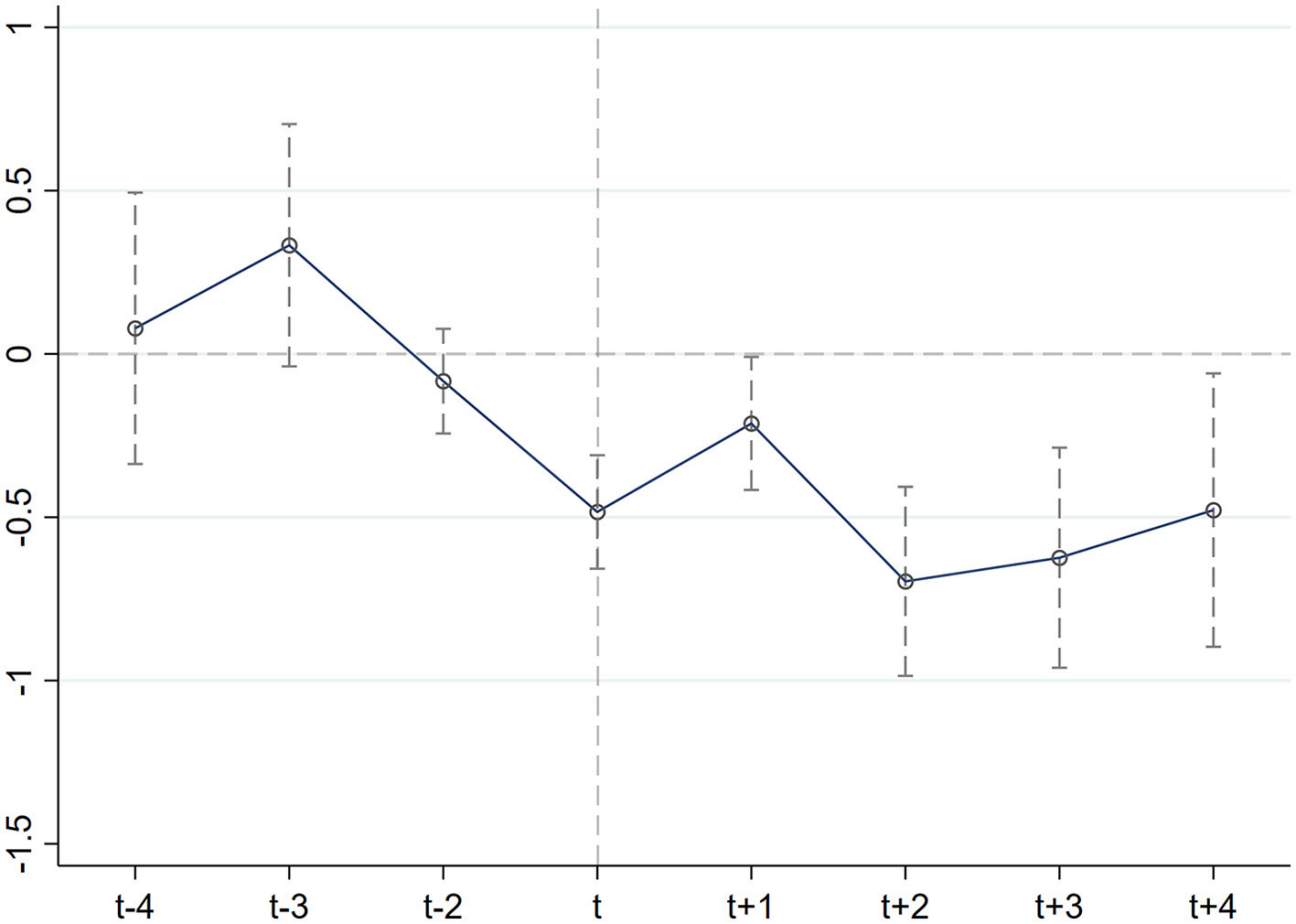
Plot of parallel trend test results.

Figure 1 presents the results of the parallel trend test. The estimation results prior to implementing Broadband China were not significant. This result shows that prior to the introduction of Broadband China, there was no systematic difference between the treatment group and the control group. Since the beginning of Broadband China, the residents' healthcare expenditure in the treatment group has been significantly reduced. The sample satisfies the parallel trend assumption.

#### 4.2.2. PSM-DID method

**Figure 2:**
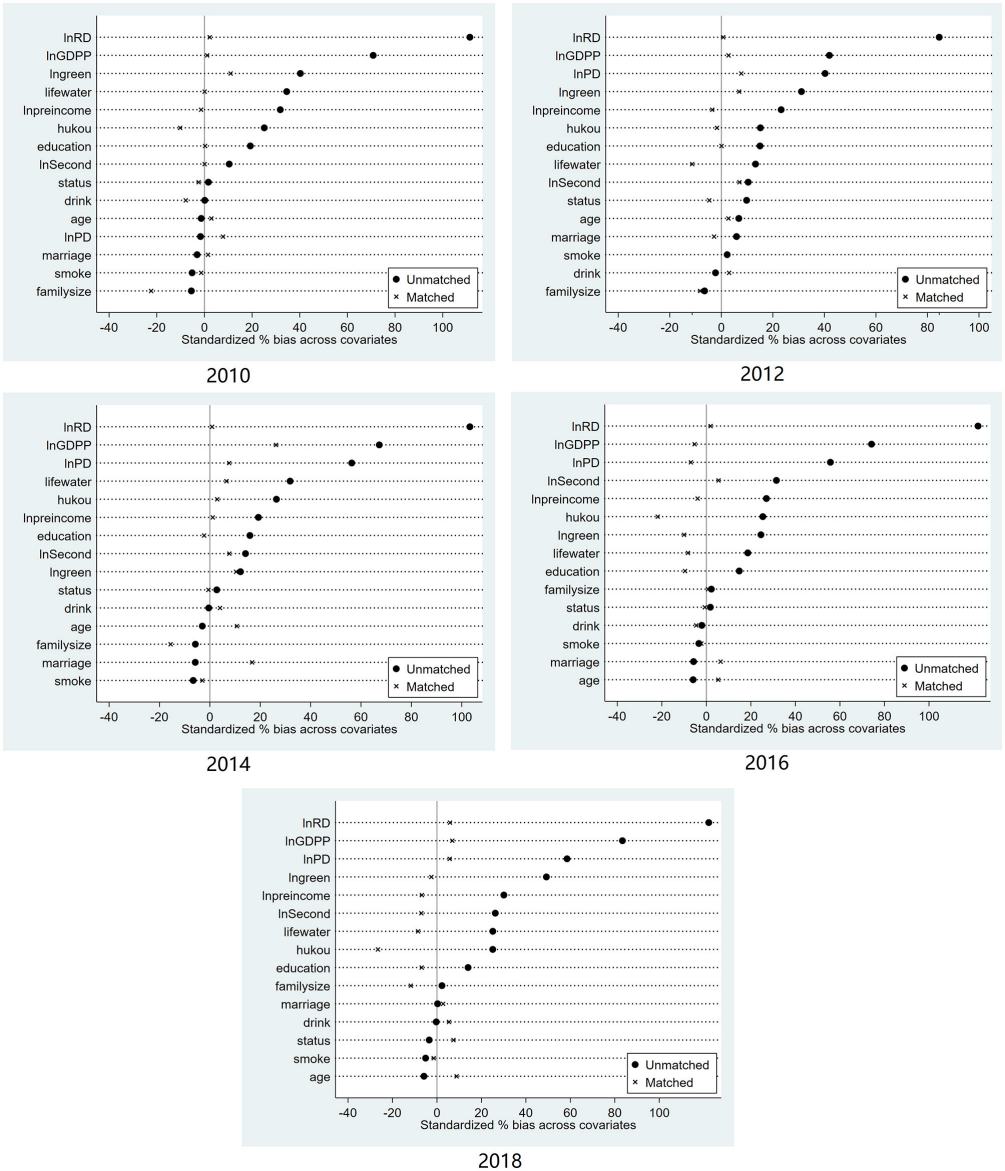
Balance test.

To overcome the systematic differences in the trends between the pilot cities of Broadband China and other cities and to reduce the estimation bias of the double difference method, this paper further uses the PSM-DID method to conduct robustness checks. Specifically, this study drew on the study by Heyman et al. to use the control variables in the benchmark regression as covariates (31). The samples were matched year by year using a matching method and then merged vertically with the matched data for each year to create a dataset that generated panel data for regression. The balance of the matching data (Figure 2) was checked. As shown in Figure 2, the deviation of the standardized mean of all matched variables after matching was <20%. This indicates that there was no systematic difference between the treatment group and the control group before the policy impact. The coefficient of PSM-DID in Table 3 (1) is −0.111 and significant. There is no significant difference when compared with the benchmark regression results, which further supports the empirical conclusion that the implementation of digital infrastructure has significantly reduced residents' healthcare expenditures.

**Table 3:** Robustness test results.

**Variables**	**ME**				
	**(1)**	**(2)**	**(3)**	**(4)**	**(5)**
Policy	−0.111^**^ (0.047)	−0.193^***^ (0.039)	−0.187^***^ (0.040)		−0.183^***^ (0.039)
Bigdata		−0.104^***^ (0.039)			
Smartcity			−0.008 (0.039)		
Fakepolicy				0.016 (0.388)	
Constant	1.163 (2.285)	−0.666 (2.115)	−1.096 (2.113)	2.392 (2.858)	2.359 (1.993)
Controls	Yes	Yes	Yes	Yes	Yes
Individual FEs	Yes	Yes	Yes	Yes	Yes
Province FEs	Yes	Yes	Yes	Yes	Yes
Year FEs	Yes	Yes	Yes	Yes	Yes
Observations	56,053	78,601	78,601	32,799	77,734
R-squared	0.023	0.023	0.023	0.022	0.023

In parentheses are standard errors adjusted for heteroskedasticity and clustering by both individual and year. ^*^, ^**^, and ^***^ are significant at the levels of 10, 5, 1%, respectively.

#### 4.2.3. Controlling other policies

Some other policies may exist that affect healthcare expenditure when Broadband China is implemented. The Broadband China dummy variable in the baseline regression model may include other policy shocks, which may lead to bias in the estimation results. We checked and selected some other policies to test whether they affect the effect of Broadband China. First, the Smart City Policy was proposed in 2012. Through a series of measures, this policy aimed to provide both a better living standard and better working services for citizens, create a more favorable business development environment for enterprises, and optimize the government with more efficient operation and management mechanisms. Among them, smart healthcare construction may also be beneficial in reducing healthcare expenditures for residents. Second, in 2016, China's National Development and Reform Commission, the Ministry of Industry and Information Technology, and the Central Internet Information Office issued a letter approving the establishment of a national-level comprehensive big data pilot zone. The Big Data Pilot Zone carries out systematic experiments around seven major tasks, including data resource management and sharing and opening, data center integration, data resource application, data element circulation, big data industry clustering, and big data system innovation. The policy may have an impact on residents' healthcare expenditures. Based on these considerations and to mitigate the potential impact of the Smart Cities Policy and Big Data pilot zone on the estimated results, we set the dummy variables for the Smart Cities in 2012, 2013, and 2014 and the Big Data pilot zone in 2016, respectively. We then introduced them into the baseline model together with Broadband China. Columns 1–3 of Table 3 show the results. The estimates show that Broadband China policy still significantly reduces healthcare expenditures after controlling for potential policy disruptions of smart cities and big data plot zones.

#### 4.2.4. Counterfactual tests

The use of the DID model requires that the treatment and control groups be comparable. Without the implementation of the digital infrastructure, there would have been no significant difference in healthcare expenditure between the treatment and control groups due to changes over time. However, in addition to broadband in China, some other policies or random factors may also cause differences in healthcare expenditures. Although these differences are not associated with the construction of broadband in China, they may ultimately contribute to the conclusions drawn in the previous section. To rule out this possibility, we applied a strategy of changing the time by drawing on the method of Rao et al. (32). We used a sham experiment with a hypothetical policy shock in 2012 to examine whether those healthcare expenditures also differed between the treatment and control groups before and after 2012. Finding a significant negative effect empirically meant that the previous regression was not meaningful. Column 4 of Table 3 shows the estimated coefficient is not significant and also suggests that the results of the baseline regressions are not due to regular random factors.

#### 4.2.5. Excluding first-tier cities

Due to the high level of digital infrastructure construction in the four first-tier cities of Beijing, Shanghai, Guangzhou, and Shenzhen, not only are their urban patterns and economic levels different from those of other cities, but there are also differences in economic decision-making and urban planning. Drawing on the method by Wu et al. (33), we excluded the samples from the four first-tier cities. Column 5 of Table 3 shows that the estimated coefficient is also significant and that digital infrastructure can still reduce residents' healthcare expenditures.

### 4.3. Heterogeneity analysis

#### 4.3.1. Residents' age

Age has long been regarded as one of the critical factors in healthcare expenditure (34). We divided ages into three categories according to the World Health Organization (WHO) criteria, namely the young group (16–44 years old), the middle-aged group (45–60 years old), and the older adult group (over 60 years old). Excluding the age variable from the baseline regression model, the regression was estimated separately for each group. The results of the estimations in Panel A of Table 4 indicate that digital infrastructure has no significant effect on the medical expenditure of residents under 44 years old and over 60 years old, while it has a significant negative effect on residents aged 45–60 years old. The reason for the result is that residents under 44 years generally have better health and lower healthcare expenditures, while people over 60 years have limited ability to benefit from digital infrastructure due to technological barriers. In contrast, for residents aged 45–60 years, medical expenditure tends to increase with age, making the digital infrastructure a significant negative factor impacting their healthcare expenditure level.

**Table 4:** Results of heterogeneity analysis.

**Panel A: age**				
**Variables**	**HE**			
	**(1) 16–44**	**(2) 45–60**	**(3)** >**60**	
Policy	−0.075 (0.065)	−0.382^***^ (0.076)	−0.077 (0.084)	
Constant	−5.419^***^ (2.031)	−4.230^**^ (1.819)	2.203 (1.961)	
Controls	Yes	Yes	Yes	
Individual FEs	Yes	Yes	Yes	
Province FEs	Yes	Yes	Yes	
Year FEs	Yes	Yes	Yes	
Observations	34,986	25,869	17,747	
R-squared	0.023	0.03	0.026	
**Panel B: education**				
**Variables**	**HE**			
	**(1)**<**6**	**(2) 6–9**	**(3) 9–12**	**(4)** >**12**
Policy	0.207^***^ (0.062)	−0.245^***^ (0.069)	−0.212^**^ (0.101)	0.118 (0.129)
Constant	−8.911^***^ (2.339)	10.830^***^ (4.199)	8.789^**^ (3.482)	1.572 (3.899)
Controls	Yes	Yes	Yes	Yes
Individual FEs	Yes	Yes	Yes	Yes
Province FEs	Yes	Yes	Yes	Yes
Year FEs	Yes	Yes	Yes	Yes
Observations	30,979	26,339	13,211	10,350
R-squared	0.026	0.022	0.033	0.036
**Panel C: income level and region**				
**Variables**	**HE**			
	**(1) Low-income**	**(2) High-income**	**(3) Urban**	**(4) Rural**
Policy	0.156^**^ (0.071)	−0.105^*^ (0.058)	−0.272^***^ (0.046)	0.026 (0.076)
Constant	0.448 (2.160)	3.104 (3.352)	0.289 (2.296)	2.065 (4.180)
Control	Yes	Yes	Yes	Yes
Individual FEs	Yes	Yes	Yes	Yes
Province FEs	Yes	Yes	Yes	Yes
Year FEs	Yes	Yes	Yes	Yes
Observations	39,183	45,067	57,125	21,476
R-squared	0.023	0.026	0.024	0.024

In parentheses are standard errors adjusted for heteroskedasticity and clustering by both individual and year. ^*^, ^**^, and ^***^ are significant at the levels of 10, 5, 1%, respectively.

#### 4.3.2. Residents' educational levels

Educational level is an important factor that affects healthcare expenditures. To account for potential heterogeneity in healthcare expenditure among residents with different educational levels, this study divided the residents into four groups based on their education level: low education level (6 years and below), a medium-low education level (6–9 years), a medium-high education level (9–12 years), and high education level (12 years and above). Panel B of Table 4 shows the heterogeneous estimated results of healthcare expenditure. The effect of digital infrastructure is significant for residents with an education level below 12 years. The above results indicate that digital infrastructure provides more online platforms and information channels, making it easier for residents with low, medium-low, and medium-high education levels to acquire health knowledge to protect themselves against diseases and thus reduce medical expenses. Therefore, the digital infrastructure plays a significantly larger role in groups with low, medium-low, and medium-high education levels.

#### 4.3.3. Residents' income levels

We analyzed the heterogeneity of income levels. Columns 1–2 in Panel C of Table 4 show that digital infrastructure has a significant negative effect on the healthcare expenditures of both low- and high-income residents. However, there is a noticeable difference in effectiveness between the two income groups, even though both remain significant at the conventional level. There are two possible reasons for the above heterogeneous results by income. First, residents with high incomes may pay more attention to healthcare and take more preventive actions to avoid risk than those with low incomes. Therefore, they are less affected by the digital infrastructure. Second, compared with the high-income group, digital infrastructure may increase low-income individuals' awareness of healthcare protection, leading them to be more affected by the construction of digital infrastructure.

#### 4.3.4. Residents' Hukou

Considering the development differences across urban and rural areas, we further examined whether the effect of healthcare expenditure varies across different types of Hukou. The samples were divided into two groups: urban and rural. Columns 3–4 in Panel C of Table 4 present the estimated results for Hukou, showing that rural Hukou healthcare expenditure is not significantly negatively affected by digital infrastructure. However, in the case of urban Hukou, digital infrastructure may lead to lower healthcare expenditures for people. In summary, the above results also suggest that in urban regions with higher levels of digital infrastructure, digital infrastructure greatly benefits people's healthcare expenditures.

Overall, the heterogeneous effects of broadband vary by age, education, income, and region. These effects are mainly observed in middle-aged urban residents and people with low income and educational levels.

### 4.4. Mechanism analysis

What mechanisms explain the digital infrastructure and the reduction in residents' healthcare expenditures? In this section, we explored two channels through which digital infrastructure reduces the healthcare expenditures of residents: the accessibility of purchasing commercial insurance and the residents' health.

#### 4.4.1. Accessibility of purchasing commercial insurance

Not only has digital infrastructure accelerated the application of new-generation digital technology, such as big data, artificial intelligence, and cloud computing, in many fields, but Internet insurance has also gradually emerged and become popular. Compared with traditional insurance, Internet insurance has the characteristics of convenience, timeliness, efficiency, innovation, and a small amount of high frequency, all of which can effectively reduce both transaction costs and the asymmetry of insurance information, breaking the spatial distance limitations and increasing the accessibility of commercial insurance for residents (35). Moreover, premium income from personal insurance (such as health, life, and accident insurance) accounts for over 80% of China's commercial insurance premium income. This indicates that commercial insurance is positively correlated with health insurance, which will reduce residents' healthcare expenditures. Therefore, digital infrastructure can reduce residents' healthcare expenditures by increasing the availability of commercial insurance.

#### 4.4.2. Residents' health

A number of relevant studies confirm that Internet users have better physical and mental health (36–40) and that health is closely related to healthcare costs (41). Hence, we argued that digital infrastructure may reduce healthcare expenditure by improving residents' health. First, digital infrastructure promotes the utilization of the Internet in the medical field, improving residents' health by alleviating the uneven allocation of medical resources and improving treatment efficiency and medical services (42, 43). For instance, the application of real-time and virtual dialogue digital technology breaks the time and space constraints of medical services and reduces irrational medical treatment behavior, which not only improves residents' own health but also reduces medical expenses. Second, digital infrastructure is conducive to popularizing health knowledge (44), which helps residents choose healthier lifestyles, improve their own health (45), and ultimately reduce medical expenses.

Based on the analysis of the theoretical mechanism, this study considered the accessibility of purchasing commercial insurance and residents' health to be the mechanism variables for the digital infrastructure and residents' healthcare expenditure. Drawing on Chen et al. (45), we constructed the following model to validate the study mechanism.


(3) $$\begin{array}{l} M_{i,j,t}=\beta_{0}+\beta_{1}{Policy}_{i,j,t}+\sum \gamma_{j}X_{i,j,t}+\mu_{j}+\eta_{j}+\upsilon_{t}+\varepsilon_{i,j,t}, \end{array}$$

where the mediating variable *M*_*i, j, t*_ is a potential mechanism variable. The signs and significance of β_1_ is the focus of this study.

Column 1 of Table 5 shows that the regression coefficient of digital infrastructure strategy on commercial insurance purchase is 0.039, which is significant at the 1% level. This suggests that network infrastructure construction increases residents' accessibility to purchasing commercial insurance, reducing their healthcare expenditure. The results in column 2 of Table 5 show that the regression coefficient of the digital infrastructure is 0.016, which is significant at the 10% level, indicating that the network infrastructure significantly improves the health level of the residents and then reduces healthcare expenditures.

**Table 5:** Mechanism analysis.

**Variables**	**Insurance**	**Health**
	**(1)**	**(2)**
Policy	0.039^***^ (0.006)	0.016^*^ (0.009)
Constant	0.462^*^ (0.251)	2.083^***^ (0.445)
Controls	Yes	Yes
Individual FEs	Yes	Yes
Province FEs	Yes	Yes
Year FEs	Yes	Yes
Observations	79,111	79,103
R-squared	0.165	0.060

In parentheses are standard errors adjusted for heteroskedasticity and clustering by both individual and year. ^*^, ^**^, and ^***^ are significant at the levels of 10, 5, 1%, respectively.

### 4.5. Social benefit analysis

The above empirical study demonstrates that digital infrastructure development significantly reduces residents' healthcare expenditures by increasing the accessibility of commercial insurance and enhancing the health of the population. In this section, we take a step further to estimate the social benefits.

Following Liao et al. (23) and Chen et al. (45), a cost-benefit analysis was conducted in this section to explore the total social costs and welfare benefits caused by digital infrastructure. The estimates in Table 2 suggest that the implementation of digital infrastructure reduces healthcare expenditures by 18.8 percent. Thus, the total social welfare benefit led by the digital infrastructure can be calculated by multiplying the estimated effect of digital infrastructure on healthcare expenditure by the total population size in China for each year and the annual average personal healthcare expenditure. For example, the product of the average healthcare expenditure (1,307 RMB), the total population size in 2016 (1.38 billion), and the estimated effect of digital infrastructure on healthcare expenditure (18.8%) is ~325 million RMB. This means that digital infrastructure construction has reduced healthcare expenditure for society by 325.3 million RMB (or 46.9 million USD). Using a similar method, we can calculate that the construction of digital infrastructure reduced social healthcare expenditure by ~419 million RMB (or 63.4 million USD) in 2018. We observed that the social benefits are becoming larger over time.

There should be other indirect social benefits except for the above direct social benefits. Since digital infrastructure plays a basic role in a digital society, the construction of digital infrastructure also has sweeping impacts on many aspects of society. For example, through online education, remote families living in some mountainous areas can get more quality education, improving human resources and bringing economic results such as a higher household income. Given the lack of relevant data, it is challenging to calculate such indirect benefits in healthcare. However, this area deserves research in the future.

## 5. Conclusion and implications

Given the context of rapid digital infrastructure construction and rising healthcare expenditure, it is important to leverage the potential of digital infrastructure to reduce healthcare expenditure and further improve residents' quality of life. Taking advantage of the quasi-natural experiment provided by the Broadband China policy and using a sample of CFPS from 2010 to 2018, our DID models show that digital infrastructure leads to a decrease in residents' healthcare expenditure.

The main conclusions are as follows. First, we found that digital infrastructure construction significantly reduces residents' healthcare expenditures. Compared with non-Broadband China pilot cities, the residents in Broadband China pilot cities reduced their healthcare expenditures by 18.8%, illustrating the apparent impact of sweeping digital technology advances on the residents' healthcare behavior. Second, the heterogeneous results show that the effects of digital infrastructure on reducing healthcare expenditure are higher for middle-aged residents, those with lower levels of education and low income, as well as those living in urban areas. Third, healthcare expenditure is influenced by digital infrastructure through the two underlying mechanisms of commercial insurance accessibility of purchasing and residents' health. Finally, we calculated the social welfare brought about by digital infrastructure. We estimated that the construction of digital infrastructure could reduce social healthcare expenditures by ~419 million RMB, or 63.4 million USD, at 2018 exchange rates.

Although our research was based on Chinese data, it has worldwide implications. In contemporary times, finding new solutions to address social healthcare issues is urgent. Governments in both developed and developing countries often face financial constraints, especially in the current era of the pandemic, inflation, and other uncertainties. This research provided new ideas for overcoming these challenges.

Several policy implications can be derived from the results of this study. First, the government should accelerate the construction of digital infrastructure and promote the widespread application of digital technologies such as 5G in the healthcare sector. Digital infrastructure construction is a key influencing factor in the development of smart healthcare, which provides diverse access to medical treatment, improves treatment efficiency, reduces unreasonable medical practices, and ultimately reduces medical expenditures. This provides a solution for developing countries to achieve good health and wellbeing through sustainable development goals. Second, the government should be aware of the potential digital divide resulting from the construction of digital infrastructure. Efforts should be made to narrow this divide by improving the digital literacy of less educated individuals and older adults and by increasing investment in the construction of digital infrastructure in less developed areas such as rural areas. This has significant implications for the digitalization of developing countries. Finally, the government should encourage insurance companies to use both the Internet and digital technology to provide diversified health insurance products, simplify the insurance purchase and claims process, effectively perform the insurance protection function, and ultimately lead to a reduction in healthcare expenses.

Despite these strengths, our study also has some limitations. First, due to data restrictions, we used the “Broadband China” policy as a proxy variable for digital infrastructure development, instead of directly measuring digital infrastructure development in cities. Second, this study measures the level of healthcare expenditure using total healthcare expenditure without differentiating between out-of-pocket and reimbursement costs. Future studies could use more detailed data to investigate the impact of digital infrastructure development on healthcare expenditures.

## Data availability statement

The original contributions presented in the study are included in the article/supplementary material, further inquiries can be directed to the corresponding authors.

## Author contributions

Conceptualization, methodology, software, validation, resources, data curation, writing—original draft preparation, and literature collection: CH. Writing—review and editing: HH. Supervision: HH, TW, and NZ. All authors have read and agreed to the published version of the manuscript.
